# Case Report: Fatal atrioesophageal fistula following atrial fibrillation ablation—critical reflections on prevention

**DOI:** 10.3389/fcvm.2025.1493259

**Published:** 2025-02-25

**Authors:** Qi Dai, Shutong Chen, Ye Yuan, Yinghao Du, Kuixin Fan, Jingfeng Zhang, Jianjun Zheng

**Affiliations:** ^1^ Department of Radiology, Ningbo No.2 Hospital, Ningbo, China; ^2^ School of Medical Imaging, Hangzhou Medical College, Hangzhou, China; ^3^ Department of Emergency Medicine, Ningbo No.2 Hospital, Ningbo, China

**Keywords:** atrioesophageal fistula, ischemic stroke, myocardial infarction, atrial fibrillation, radiofrequency ablation

## Abstract

Radiofrequency ablation (RFA) is an important therapeutic modality for atrial fibrillation (AF), widely utilized in clinical practice due to its safety and significant efficacy. However, post-procedural complications may arise, influenced by anatomical positioning and the intensity of ablation energy, with atrioesophageal fistula (AEF) being particularly rare yet severe. This case report describes a unique instance of a patient developing AEF following AF ablation, accompanied by ischemic stroke and myocardial infarction. A 71-year-old male admitted to the emergency department on July 19, 2024, with acute loss of consciousness and convulsions. Upon admission, physical examination and laboratory tests revealed vital signs within abnormal ranges and indicators suggesting inflammation and potential myocardial injury. Head CT scans showed hypoattenuating areas indicative of cerebral infarction, chest CT suggested possible air accumulation in the left atrial region. ECG findings were consistent with atrial flutter, myocardial infarction, and incomplete right bundle branch block. Given his history of atrial fibrillation and RFA, alongside clinical manifestations, the patient was diagnosed with cardio-cerebral syndrome, suspected to be complicated by an AEF due to the presence of air in the left atrium. AEF diagnosis was confirmed via cardiac CTA, leading to conservative management decisions. Despite initiating thrombolysis for cerebral infarction and supportive treatments for heart failure, including VA-ECMO, the patient's condition continued to decline, evidenced by cardiogenic shock, heart failure, and progressive neurological deficits including coma and dilated non-reactive pupils. Ultimately resulting in family-elected discharge against medical advice on the fourth day of hospitalization.

## Introduction

Atrioesophageal fistula (AEF) is an exceedingly rare but life-threatening complication following radiofrequency ablation (RFA) for atrial fibrillation (AF). RFA involves the targeted application of thermal energy to disrupt abnormal electrical pathways in the heart, aiming to restore normal sinus rhythm. The procedure typically includes catheter insertion through the femoral vein or artery and navigation to the left atrium under fluoroscopic guidance. Despite its high efficacy and relative safety, complications such as AEF can arise due to anatomical proximity and variations, where excessive heat may damage the esophagus adjacent to the left atrium. Notably, concomitant ischemic stroke (IS) and myocardial infarction (MI) can occur postoperatively, further complicating the clinical picture. These conditions, if not managed aggressively, can significantly increase mortality. The onset of AEF can be delayed by days to weeks after the RFA procedure, with initial symptoms lacking specificity. Combined with rapid disease progression, this makes diagnosis challenging.

In this case, a patient presented with nonspecific neurological signs six hours after the onset of acute loss of consciousness and convulsions, occurring approximately one month after RFA. Upon hospital admission, the patient exhibited elevated troponin levels indicative of MI and imaging findings suggestive of cerebral infarction and gas accumulation in the left atrium. Continuous imaging follow-up and laboratory tests are essential for auxiliary diagnosis. In this instance, cranial and thoracic computed tomography (CT) scans revealed a hypodense area in the right parietal lobe consistent with cerebral infarction and evidence of gas in the left atrial region, leading to a diagnosis of AEF. Given that the patient was within the therapeutic window for stroke, thrombolytic therapy was administered. However, despite these interventions, the patient's condition deteriorated, marked by cardiogenic shock and progressive neurological deficits. However, Surgical repair should be promptly performed once AEF is confirmed, highlighting the importance of early detection and timely intervention.

## Case

A 71-year-old male was admitted to the emergency department on July 19, 2024, presenting with acute loss of consciousness and convulsions lasting six hours. The patient experienced a sudden onset of altered mental status, unresponsiveness, and vomiting at home without any apparent precipitating factors. He vomited once, with the contents being gastric material. On admission, the patient's vital signs were as follows: pulse rate 95 beats per min, respiratory rate 18 breaths per min, blood pressure 145/63 mmHg, and temperature 37.2 °C. He was unresponsive to verbal stimuli, exhibited weak pain responses in all extremities, and had a positive Babinski sign in the right lower limb. Upon admission, laboratory investigations revealed the following results: white blood cell count 11.1 × 10^9^/L, absolute neutrophil count 10.5 × 10^9^/L, absolute lymphocyte count 0.2 × 10^9^/L, platelet count 121 × 10^9^/L, prothrombin time 13.1 s, prothrombin activity 71.5%, and D-dimer 3,583.0 ng/ml (DDU). Cardiac enzymes showed high-sensitivity troponin I 0.5374 ng/ml and B-type natriuretic peptide 106 pg/ml. Liver function tests revealed direct bilirubin 8.9 µmol/L and indirect bilirubin 16.1 µmol/L. Glucose metabolism parameters included glucose 6.88 mmol/L and lactate dehydrogenase 277 U/L.

**Figure 1:**
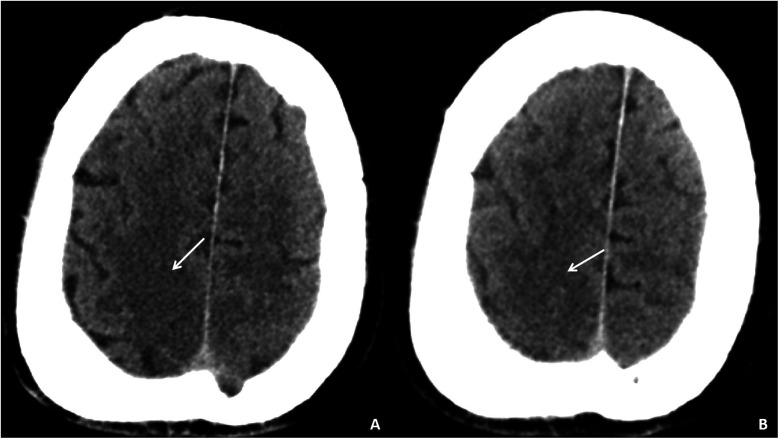
Cranial CT images of the patient upon admission. Slightly lower density areas with unclear boundaries, primarily located in the white matter region of the right parietal lobe, are visible on two different axial sections **(A,B)**.

**Figure 2:**
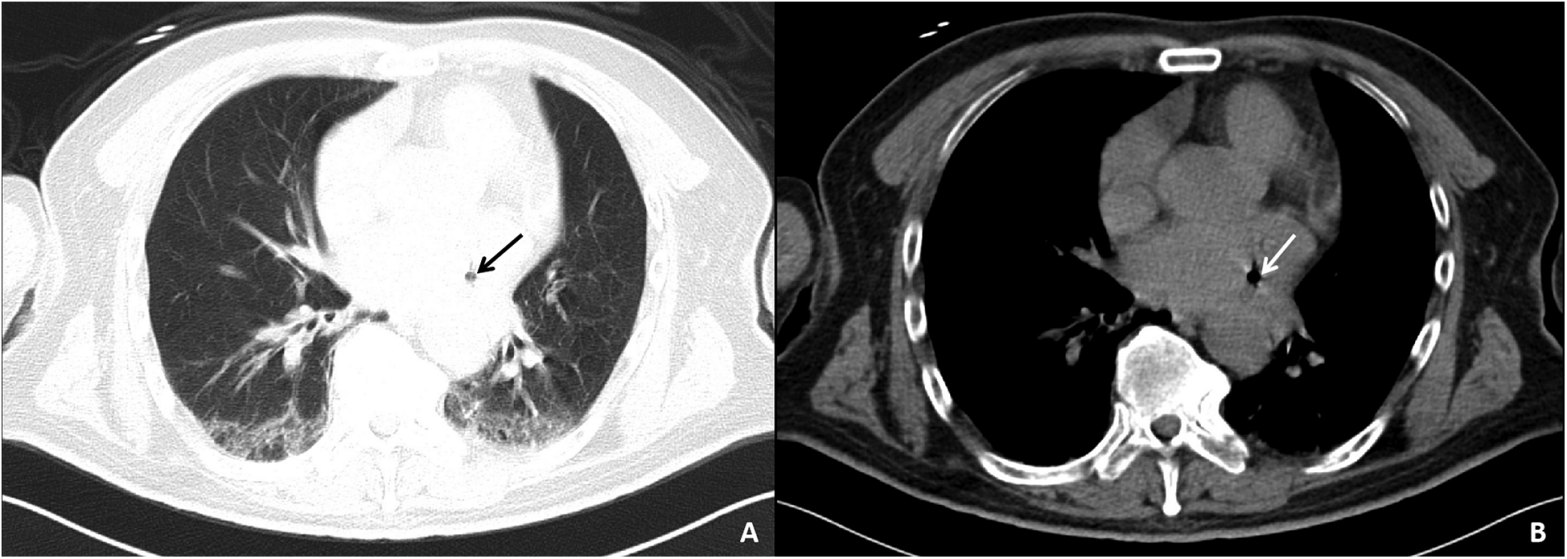
Chest CT images of the patient upon admission. The pulmonary window **(A)** and mediastinal window **(B)** both show a lucency in the area of the left atrium, suggesting a small amount of air within the left atrium.

**Figure 3:**
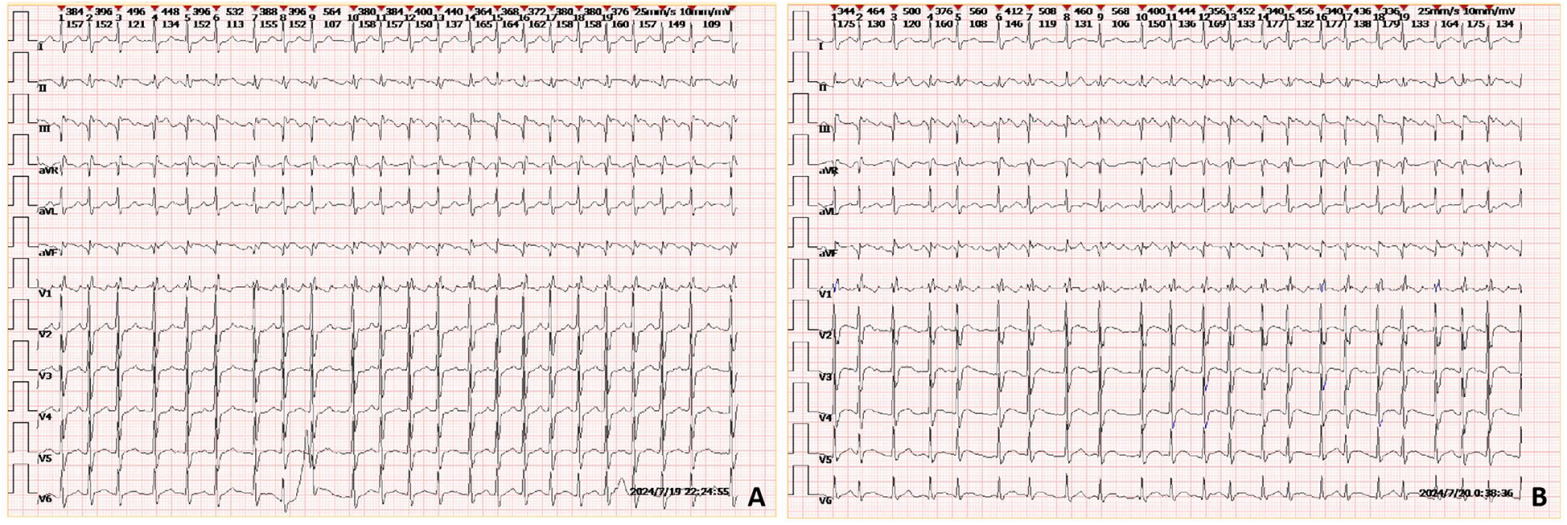
ECGs of the patient taken upon admission **(A)** and during the early morning of the second day **(B)** the ECGs show rapid atrial flutter, incomplete right bundle branch block, and ST-segment elevation with abnormal Q waves in leads II and aVF.

Auxiliary examinations included cerebral artery computed tomography angiography (CTA), which revealed cerebral atherosclerosis with mild local luminal stenosis. CT scans of the head and chest revealed a slightly hypoattenuating area in the right frontoparietal junction (Figure 1) and a radiolucent area in the left atrial region suggesting possible air accumulation (Figure 2). Specifically, the cranial CT images upon admission showed slightly lower density areas with unclear boundaries, primarily located in the white matter region of the right parietal lobe, indicative of an acute ischemic event rather than chronic changes. The presence of these hypodense areas, combined with the patient's clinical presentation and elevated troponin levels, suggested an acute ischemic stroke. These findings warranted further investigation. Electrocardiogram (ECG) findings indicated atrial flutter with rapid ventricular response, ST-segment elevation in the inferior leads with abnormal Q waves, and incomplete right bundle branch block (Figure 3A). The patient had a history of atrial fibrillation and had undergone RFA one month prior at another hospital. The provisional diagnoses included cerebral infarction, shock, coma, and heart failure. The patient's history included RFA for atrial fibrillation. As the patient was within the therapeutic window for stroke, treatment with 1 million IU of urokinase for thrombolysis was initiated with consent. Urokinase, a fibrinolytic agent effective in dissolving clots and restoring blood flow, was chosen for its balanced safety and efficacy, especially crucial for rapid reperfusion. Compared to alteplase (tPA), urokinase may reduce the risk of systemic bleeding complications in certain patients. Symptomatic and supportive measures, including fluid resuscitation and vasopressor support, were provided to improve cerebral circulation and for early secondary prevention of stroke. Continuous monitoring of cardiac and hemodynamic parameters was also conducted.

On the morning of the second day of admission, the patient remained confused and comatose, necessitating endotracheal intubation. At 01:52, laboratory results showed high-sensitivity troponin I at 4.3153 ng/ml, creatine kinase at 330 U/L, and lactate dehydrogenase at 328 U/L. An ECG revealed ST-T segment elevation with abnormal Q waves (Figure 3B). The clinical diagnosis was cerebral infarction concurrent with myocardial infarction, possibly indicating a cardio-cerebral syndrome. At 06:37, a recheck of high-sensitivity troponin I indicated a level of 14.0650 ng/ml. Coronary angiography was performed at 10:27, revealing no significant arterial stenosis. However, the clinical diagnosis remained cardio-cerebral syndrome and cerebral infarction. The patient continued to experience cardiogenic shock and acute heart failure, prompting the initiation of veno-arterial extracorporeal membrane oxygenation (VA-ECMO) treatment. VA-ECMO was chosen for its dual respiratory and circulatory support, essential for stabilizing severe cardiogenic shock. This intervention was necessary to maintain organ perfusion and improve survival chances while exploring further diagnostics and therapies.

**Figure 4:**
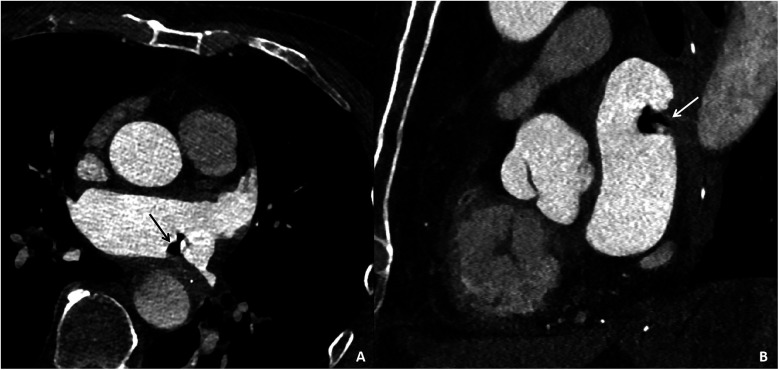
Cardiac CTA images of the patient taken on the third day of hospital admission. Contrast-enhanced CT images in the axial plane **(A)** and sagittal plane **(B)** reveal gas accumulation in the left atrial region, swelling of the adjacent esophageal wall, and obliteration of the fat planes surrounding the esophagus, confirming the presence of an esophago-left atrial fistula.

On the third day, during ward rounds, the patient was sedated with a pulse rate of 144 beats per min and a blood pressure of 78/69 mmHg. Further analysis of the patient's history revealed poor cardiac function and a critical condition. Review of the initial emergency CT scan indicated a small amount of air accumulation in the left atrial region, coupled with the recent history of radiofrequency ablation for atrial fibrillation, raising suspicion of an atrioesophageal fistula (AEF). Subsequent repeat cardiac CTA demonstrated a distinct radiolucent area within the left atrium, confirming the presence of air (Figure 4). Following multidisciplinary consultation, the patient was diagnosed with AEF, concurrent with acute myocardial infarction and cerebral infarction, likely due to air embolism. The treatment team informed the family about the patient's grave condition and, after deliberation, decided on conservative management with close and continuous monitoring of the patient's clinical status.

On the fourth day, the patient exhibited dilated pupils, measuring 4 mm in diameter, with absent light reflexes. Mannitol was administered intravenously for the purposes of reducing intracranial pressure, and conservative treatment was continued. At 15:01, the patient remained comatose with dilated, non-reactive pupils. Pain and rebound tenderness could not be assessed, and limb strength could not be evaluated. After being informed of the patient's condition, the family elected to discharge the patient against medical advice. VA-ECMO support was withdrawn, and discharge procedures were initiated.

## Discussion

AEF is a rare but serious complication following RFA for AF. According to limited reports, the incidence ranges from 0.02% to 1.5%, with a mortality rate as high as 40%–80% (1–3). The formation of RFA-induced injuries results from resistive heating and conductive heating (4). Due to the anatomical proximity of the left atrium and the esophagus, resistive heating during the procedure can directly damage the esophageal mucosa. Conductive heating spreads the ablation energy to surrounding tissues, potentially affecting the esophagus, blood vessels, and neural plexuses, which may indirectly lead to esophageal wall perforation and subsequently cause an AEF.

AEF predominantly manifests between 1 and 6 weeks post-procedure, with approximately 80.82% of cases occurring within 30 days of ablation (5). In an international multicentre registry analyzing 553,729 atrial fibrillation catheter ablation procedures across 35 countries (6), the overall incidence of oesophageal fistula was reported to be 0.025% (0.038% for radiofrequency and 0.0015% for cryoballoon, *P* < 0.0001). Symptoms typically manifested after a median of 18 days, with diagnosis confirmed at a median of 21 days post-ablation. These findings underscore the rare but significant risk of oesophageal fistula following catheter ablation, highlighting notable differences in incidence between different ablation modalities. Studies have also shown that around 40% of patients may exhibit asymptomatic esophageal lesions detectable via endoscopy, with deep ulcers considered a precursor to AEF development (7). The clinical symptoms of AEF are typically non-specific and initially may present as fever, chest pain, recurrence of atrial fibrillation or atrial flutter, loss of consciousness, and neurological signs such as stroke and seizures (1). Given that these signs can be overshadowed by symptoms of cerebral infarction and are often atypical, it is crucial to consider AEF in the differential diagnosis, especially when patients present with atypical neurological symptoms post-ablation. Differential diagnosis should involve careful consideration of alternative causes, including ischemic stroke, hemorrhagic stroke, and other potential complications of cardiac procedures.

However, AEF is frequently missed, with an early diagnosis rate of only 35% (3). Strategies to avoid overlooking AEF include maintaining a high index of suspicion, particularly in patients presenting with new-onset neurological deficits within days to weeks after catheter ablation. Clinicians should conduct thorough evaluations, including imaging studies like chest enhanced CT scans and endoscopic examinations, to identify potential esophageal injuries. Early recognition of subtle signs such as unexplained fever or chest pain in the context of recent ablation can prompt further investigation and timely intervention. In the current case, the primary clinical manifestations and diagnosis were cerebral infarction and myocardial infarction, with the patient's surgical history overlooked, leading to a missed diagnosis. Furthermore, late-onset complications can include stroke, cerebral hemorrhage, sepsis, coma, acute heart failure, cardiac tamponade, and gastrointestinal bleeding. By this stage, most patients who have undergone ablation have been discharged, and if timely diagnosis and treatment are not provided within the therapeutic window, these conditions can pose a severe threat to the patient's life.

Due to the rarity of AEF cases, there are currently no formal guidelines for diagnostic methods. Diagnosis primarily relies on a combination of imaging studies, including cranial and thoracic CT, coronary angiography, echocardiography, and endoscopic examination of the digestive tract (8). One study reported that among 118 confirmed cases of AEF, 80.2% were identified through thoracic CT, which revealed a pathological fistulous tract between the esophagus and the left atrium, and a distinct radiolucent area in the atrial region (6). Additionally, 20.7% of cases were diagnosed via endoscopy; however, due to the nonspecific nature of the initial signs, many patients did not undergo endoscopic examination, thereby increasing the difficulty of diagnosis. Furthermore, cranial CT or magnetic resonance (MR) imaging in these patients often shows ischemic lesions, suggesting transient gas emboli entering the cerebral arteries, which must be differentiated from isolated cerebrovascular accidents and cerebral infarctions. A European cardiology journal noted that the average time to diagnose AEF is around three days, with the longest delay reaching up to 42 days (9). This delay is attributed to the initial nonspecific signs, subtle cardiac symptoms, and the delayed onset of postoperative complications. Given the complexity and rarity of this condition, and considering the concurrent occurrence of cerebral stroke and myocardial infarction in this case, rapid diagnosis remains challenging. Continuous imaging, laboratory tests, clinical signs, and a history of atrial fibrillation are essential for the early detection of AEF.

The final diagnosis of AEF in this case was based on the following criteria: (1) elevated white blood cell count upon admission, indicating infection; (2) a history of RFA for AF one month prior, within the typical timeframe for AEF onset; (3) cerebral CT showing ischemic changes, ECG demonstrating ST-segment elevation and abnormal Q waves, along with persistently elevated troponin levels, confirming cerebral infarction and myocardial infarction, which were likely caused by transient embolization due to air in the left atrium, supporting the diagnosis of AEF; (4) thoracic CT and cardiac CTA directly revealing air accumulation in the left atrium, confirming the suspicion of AEF. Given the severity and rapid deterioration of the patient's condition, compounded by the co-occurrence of ischemic stroke and myocardial infarction, clinicians failed to promptly integrate the CT findings with the patient's surgical history for an early diagnosis, ultimately opting for supportive care. Therefore, healthcare providers across specialties must remain vigilant regarding the possibility of such conditions, emphasizing the importance of a thorough review of the medical history and imaging studies, and implementing preventive measures preemptively.

Several clinical trials have attempted to improve RFA procedures for AF to prevent the occurrence of AEF. Kronenberger et al. (10) demonstrated that using an insulated multi-sensory intraluminal esophageal temperature probe during AF RFA aids in confirming correct catheter positioning and safe energy application, reducing esophageal thermal injuries during RFA. Esophageal cooling techniques (11) further mitigate the severity of thermal lesions caused by RFA. Intraoperative visualization of the esophagus (12) helps avoid contact between the ablation catheter and adjacent tissues, minimizing the risk of thermal injury. Weiss et al. (13) introduced an esophageal deflector combined with rigorous esophageal temperature monitoring, resulting in a 5.7% reduction in esophageal mucosal ablation injuries. Pulsed field ablation (PFA), employing non-thermal electric fields for irreversible electroporation, represents a significant advancement in interventional electrophysiology (14). PFA selectively disrupts myocardial cell membranes without causing collateral damage, achieving rapid pulmonary vein isolation with minimal complications. Clinical studies show reduced procedure times and lower incidences of esophageal and phrenic nerve damage compared to traditional RFA or cryoablation (15).

To prevent AEF, integrating low-risk and cost-effective measures during ablation is essential. Recent advancements, such as combining highly localized impedance (LI) and contact force (CF) measurements, improve tissue characterization and lesion prediction during pulmonary vein isolation. Lepillier et al. (16) showed that LI-guided ablation with ideal drops (>21 Ω anterior, >18 Ω posterior) and higher CF significantly reduces radiofrequency delivery time. Additionally, conscious sedation may reduce esophageal injury risk compared to general anesthesia. Di Biase et al. (17) found a markedly higher rate of esophageal damage under general anesthesia (48%) than with conscious sedation (4%). In conclusion, Given the severity of postoperative complications, especially for high-risk AF patients, perioperative evaluation and prevention remain critical, adopting advanced technologies along with conscious sedation, maybe significantly reduce AEF risk and enhance patient outcomes.

The primary treatment for AEF, a rare condition, involves surgical intervention. The surgical approach typically includes a right posterolateral thoracotomy followed by left atriotomy and repair. Several case reports (18, 19) have confirmed that proactive surgical management significantly enhances patient survival rates, reinforcing the importance of early definitive diagnosis and timely treatment within the surgical window. Additionally, air embolism and septic shock, which are contributing factors to mortality in AEF cases, should be adequately addressed during the perioperative period.

## Conclusion

AEF is a rare but severe complication following RFA for AF, underscoring the paramount importance of accurate and early diagnosis. This case tragically illustrates that timely diagnosis and intervention could have potentially saved the patient's life if the condition had been identified and corrected within the first few hours of hospital admission. Therefore, enhancing awareness of AEF among clinicians across specialties is crucial, particularly for those less familiar with interventional electrophysiology. Intensified post-operative monitoring of patients undergoing catheter ablation, with an extended observation period as necessary, especially for high-risk patients with a history of cerebrovascular or coronary artery disease, is essential.

Given the critical nature of AEF, early definitive diagnosis and prompt surgical intervention are vital for life-saving outcomes. These measures require multidisciplinary collaboration, involving physicians who consider the patient's RFA history, perform precise cardiac imaging assessments, and provide close post-operative surveillance. Such comprehensive approaches aim to improve survival rates by ensuring early detection and treatment, thereby preventing, detecting, and managing this devastating complication as swiftly as possible. The failure to diagnose AEF in a timely manner and the consequent delay in appropriate surgical intervention underscore the necessity of these measures. Emphasizing the importance of immediate recognition and action can significantly enhance patient outcomes and potentially save lives.

## Data Availability

The original contributions presented in the study are included in the article/Supplementary Material, further inquiries can be directed to the corresponding authors.
